# SMARCB1 (INI-1)-deficient carcinoma with eosinophilia and cervical lymph node metastasis of unknown primary: a case report and literature review

**DOI:** 10.3389/fonc.2026.1853027

**Published:** 2026-07-08

**Authors:** Lei Zhang, Tian-yu Wang, Xue-song He, Quan Zhou, Hai-juan Lv, Rui-tao Lai, Zhe Jiang, Fen-fen Jiang

**Affiliations:** 1 Department of Geriatrics, The Second Affiliated Hospital of Jiaxing University, Jiaxing, China; 2 Department of Central Laboratory, The Second Affiliated Hospital of Jiaxing University, Jiaxing, China; 3 Department of Pathology, The Second Affiliated Hospital of Jiaxing University, Jiaxing, China; 4 Department of Radiology, The Second Affiliated Hospital of Jiaxing University, Jiaxing, China; 5 Department of Ultrasonography, The Second Affiliated Hospital of Jiaxing University, Jiaxing, China; 6 Department of Cardiology, The Affiliated Hospital of Jiaxing University, Jiaxing, China

**Keywords:** cancer of unknown primary, SMARCB1, switch/sucrose non-fermentable chromatin remodeling complexes, tumor-associated blood eosinophilia, undifferentiated carcinoma

## Abstract

**Background:**

Cancer of unknown primary (CUP) represents a heterogeneous group of metastatic malignancies in which the primary site remains unidentified despite comprehensive clinical, laboratory, radiological, and pathological evaluation. CUP is generally characterized by aggressive biological behavior, poor prognosis, and the absence of standardized treatment strategies. Tumor-associated blood eosinophilia (TABE) is relatively uncommon and typically occurs after tumor dissemination, often indicating an unfavorable prognosis. SMARCB1 (INI-1) is a core subunit of the switch/sucrose non-fermentable (SWI/SNF) chromatin remodeling complex, and its functional loss can drive tumorigenesis through epigenetic dysregulation. However, cases of SMARCB1-deficient carcinoma presenting as CUP with marked TABE are exceedingly rare, posing significant diagnostic and therapeutic challenges.

**Case report:**

A 71-year-old man presented with a painless mass in the left side of the neck accompanied by generalized pruritus. Laboratory evaluation revealed persistent peripheral blood eosinophilia, and imaging studies demonstrated multiple enlarged lymph nodes in the left supraclavicular region and abdominal cavity. Histopathological examination of a lymph node biopsy showed poorly differentiated carcinoma. Immunohistochemistry revealed loss of nuclear SMARCB1 expression. A final diagnosis of SMARCB1-deficient undifferentiated carcinoma was established, and comprehensive systemic evaluation failed to identify a primary tumor. The patient received paclitaxel combined with cisplatin and a programmed cell death protein 1 (PD-1) inhibitor. Immunotherapy was discontinued due to the development of immune-related cutaneous adverse events. Despite subsequent management, the disease progressed rapidly, with the development of brainstem metastasis, and the patient ultimately died.

**Conclusion:**

We report a case of SMARCB1-deficient undifferentiated carcinoma presenting as CUP, predominantly with cervical lymph node metastases, accompanied by marked TABE. The benefit of current empirical treatment strategies appears limited. Future prospective clinical studies targeting the epigenetic vulnerabilities of this entity are warranted to improve patient outcomes.

## Introduction

Cancer of unknown primary (CUP) refers to a diverse collection of metastatic cancers whose primary site remains unidentified despite comprehensive and systematic clinical, laboratory, radiological, and pathological evaluations (1). CUP constitutes roughly 2-5% of all human malignancies. Although its incidence has shown an overall declining trend, survival outcomes have not improved (2). Most CUP cases share several common features, including aggressive behavior, early dissemination, atypical patterns of metastasis, and poor response to empirical chemotherapy. Consequently, the prognosis for the majority of individuals with CUP is poor, with a median overall survival (OS) ranging from 7 to 11 months (3). At present, no consensus has been reached regarding the standard treatment for CUP, and management remains largely empirical, commonly consisting of broad-spectrum chemotherapy regimens such as paclitaxel and platinum-based agents (4). In addition, although several studies have investigated the application of targeted therapy or immunotherapy in patients with CUP, the survival outcomes associated with these individualized treatment approaches remain controversial. The randomized CUPISCO trial demonstrated that, among selected patients with unfavorable non-squamous CUP whose disease was controlled after platinum-based induction therapy, molecularly guided treatment improved progression-free survival compared with continued standard chemotherapy. However, because the study population was highly selected, these findings cannot be directly generalized to all patients with CUP (5). A single-arm phase II study of pembrolizumab reported an objective response rate of approximately 20% and a 27-week progression-free survival rate of 28%, suggesting that a subset of patients may benefit; however, the study was limited by its small sample size and lack of a randomized control group (6). Phase II studies of nivolumab and nivolumab plus ipilimumab similarly demonstrated antitumor activity in selected patients, although their conclusions were limited by patient selection, small sample sizes, and non-randomized study designs (7).

Hypereosinophilic dermatitis (HED) is characterized by an increased absolute eosinophil count in the peripheral blood accompanied by cutaneous involvement, and it is commonly encountered as a secondary manifestation of allergic conditions, parasitic infections, collagen vascular diseases, or drug hypersensitivity reactions (8). Persistent eosinophil activation may promote eosinophil trafficking to multiple organs, including the skin, airways, gastrointestinal tract, heart, and nervous system, and can lead to end-organ injury through thrombosis and fibrosis (9). Tumor-associated blood eosinophilia (TABE) is a rare phenomenon that typically occurs after tumor dissemination, and its presence often indicates a poor prognosis (10). SMARCB1 (INI-1), located on chromosome 22q11.2, is a core subunit of the switch/sucrose non-fermentable (SWI/SNF) chromatin-remodeling complex and plays a pivotal role in adenosine triphosphate (ATP)-dependent chromatin remodeling. SMARCB1 is ubiquitously expressed in normal cells and exerts tumor-suppressive effects by transcriptionally regulating the cell cycle, proliferation, and differentiation (11). It has been reported that SMARCB1 alterations occur in approximately 1.4% of all cancers (12). The molecular hallmarks of SMARCB1-deficient cancers include deletion and/or inactivating mutations at the SMARCB1 locus, along with complete loss of nuclear expression of SMARCB1 in neoplastic cells as demonstrated by immunohistochemistry (13). Despite substantial progress in recent years, effective therapies for patients with SMARCB1-deficient cancers remain elusive.

We reported a case of cervical lymphadenopathy accompanied by eosinophilia. Despite an extensive diagnostic workup, no obvious primary lesion was identified. The patient was diagnosed with SMARCB1-deficient undifferentiated carcinoma and was treated with paclitaxel plus cisplatin concurrently with a programmed cell death protein 1 (PD-1) inhibitor. Heterogeneous malignancies presenting initially with eosinophilia are easily overlooked and may lead to diagnostic challenges; therefore, this case provides important learning points for clinicians.

## Case report

**Figure 1:**
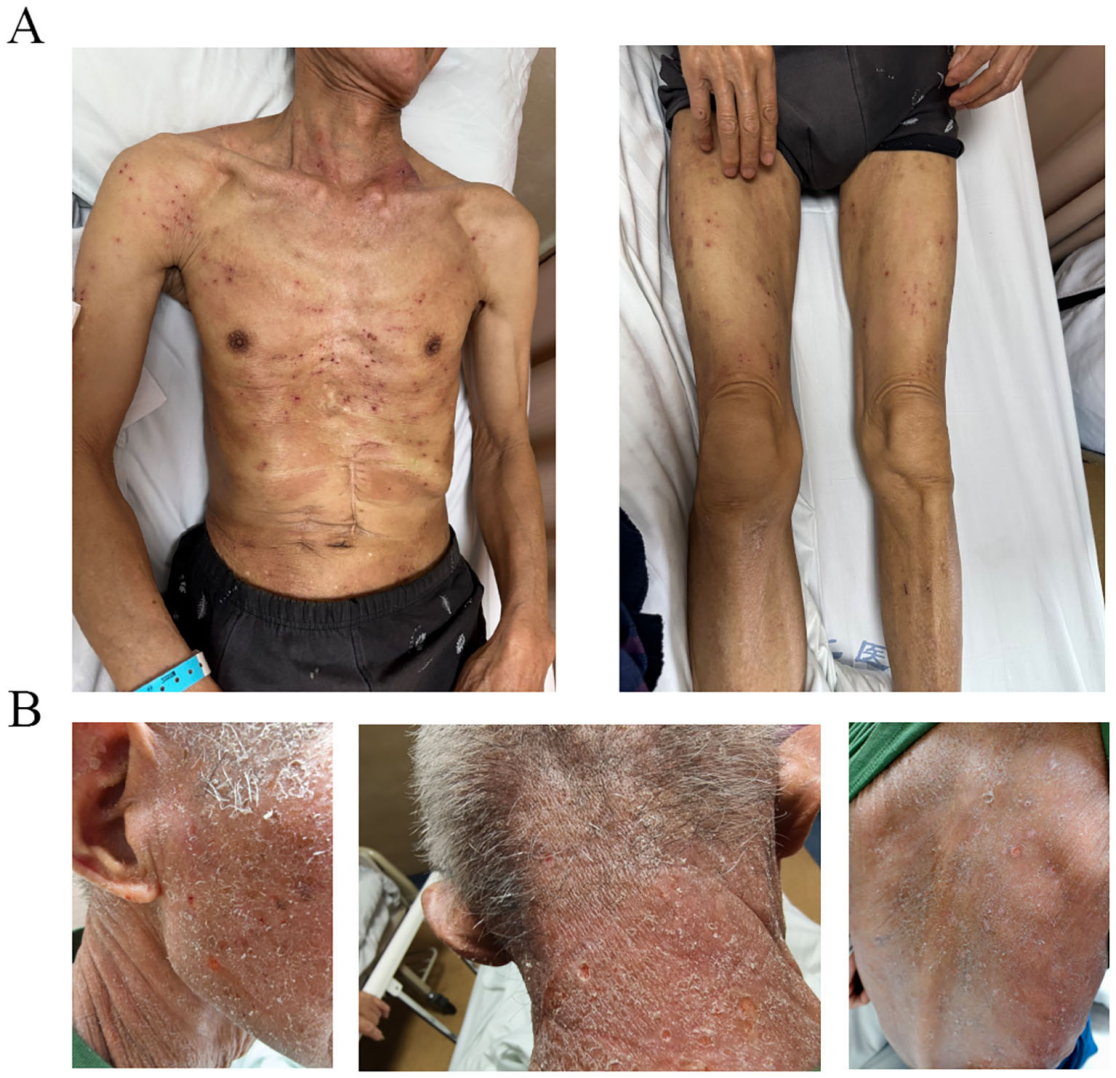
Disseminated erythematous skin lesions and erythema with scaling in the patient. **(A)** Disseminated erythematous macules over the trunk and bilateral lower limbs. **(B)** Erythema with scaling involving the face, neck, and back, indicating widespread cutaneous involvement.

**Figure 2:**
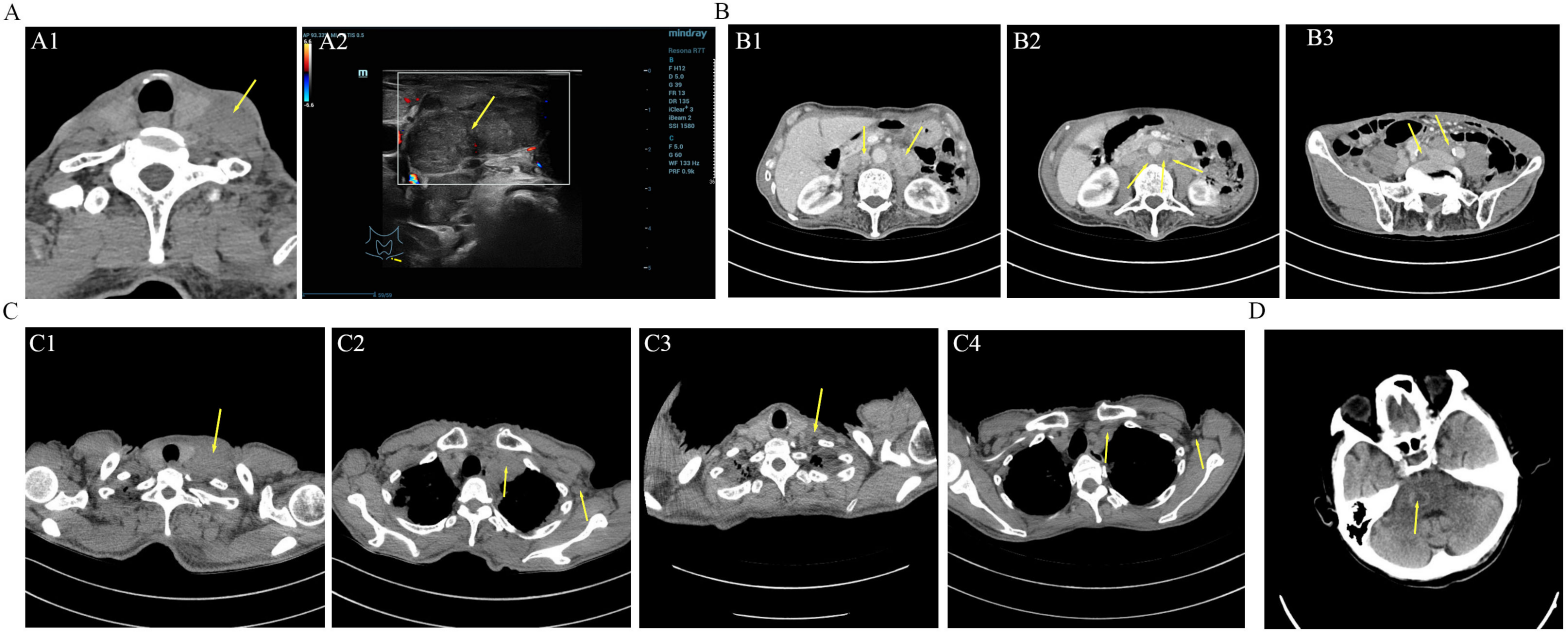
CT and ultrasound findings of lymph node metastases and brainstem involvement. **(A)** Left supraclavicular lymph node at diagnosis (CT, A1; ultrasound, A2). **(B)** Abdominal CT showing retroperitoneal and para-iliac lymph node metastases (B1-B3). **(C)** CT comparison before (May 2025; C1-C2) and after treatment (August 2025; C3-C4). **(D)** Brain CT showing brainstem metastasis (December 2025). CT, computed tomography.

**Figure 3:**
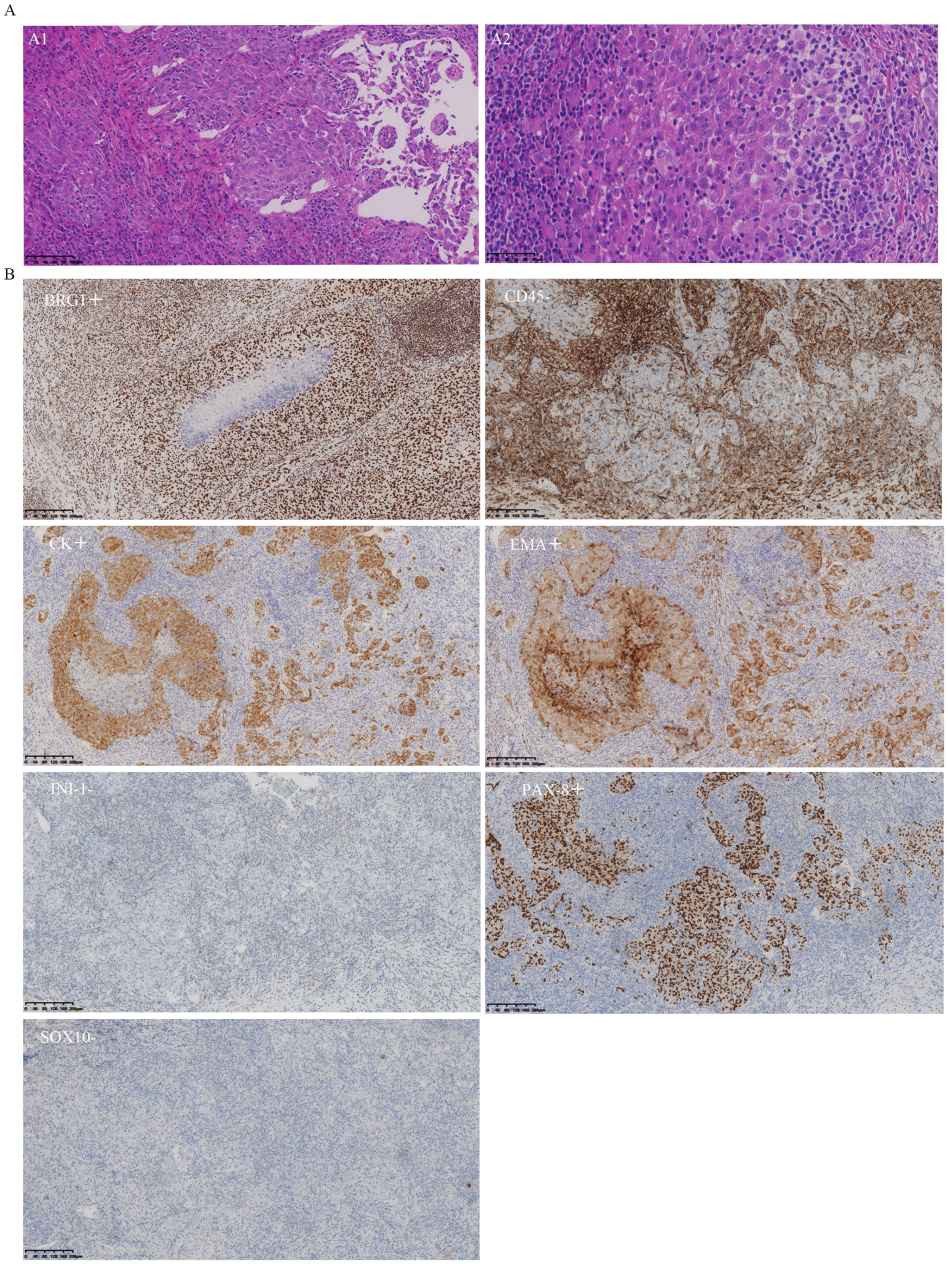
H&E and immunohistochemistry of the left supraclavicular lymph node. **(A)** H&E staining (100 μm in A1; 50 μm in A2). **(B)** Immunohistochemical staining for BRG1, CD45, CK, EMA, INI-1, PAX8, and SOX10 (200 μm).

In March 2025, a 71-year-old man presented with an asymptomatic mass on the left side of the neck accompanied by generalized pruritus. Physical examination revealed disseminated erythematous macules over the body (**Figure 1A**), with no other remarkable findings. Hematologic testing showed persistent eosinophilia. Neuron-specific enolase (NSE) was elevated (35.16 ng/mL), whereas other tumor markers, including alpha-fetoprotein (AFP), carbohydrate antigen 19-9 (CA19-9), and carcinoembryonic antigen (CEA), were within the normal ranges. The patient had been previously healthy, with no history of hypertension or diabetes. He had never smoked and reported no family history of malignancy. Cervical computed tomography (CT) revealed a mass-like soft-tissue-density lesion in the left supraclavicular region, and cervical ultrasonography demonstrated enlarged left supraclavicular lymph nodes (**Figure 2A**). No suspicious thyroid nodules or thyroid-centered lesions were identified. Subsequently, an excisional biopsy of a relatively large left supraclavicular lymph node was performed, which showed metastatic poorly differentiated carcinoma (**Figure 3A**). Immunohistochemistry showed CK and EMA positivity, loss of SMARCB1 (INI-1), retained SMARCA4 (BRG1), PAX8 positivity, and LCA (CD45) and SOX10 negativity (**Figure 3B**). Based on the morphologic features and immunophenotype, the final pathological diagnosis was SMARCB1-deficient undifferentiated carcinoma. To identify the primary lesion, the patient underwent contrast-enhanced abdominal CT, which revealed multiple enlarged intra-abdominal lymph nodes (**Figure 2B**). No focal renal mass or other suspicious renal lesion was identified, and there was no radiological evidence of a primary tumor involving the renal pelvis or other intra-abdominal organs. In addition, ultrasonography and magnetic resonance imaging (MRI), nasopharyngolaryngoscopy, upper and lower gastrointestinal endoscopy, and bone marrow aspiration and biopsy were performed; however, no primary site was detected. The clinical findings and the excisional biopsy results were suggestive of metastatic disease, yet comprehensive systemic assessment did not reveal the underlying tumor. Therefore, the malignancy was identified as CUP with distant lymph node metastases.

**Figure 4:**
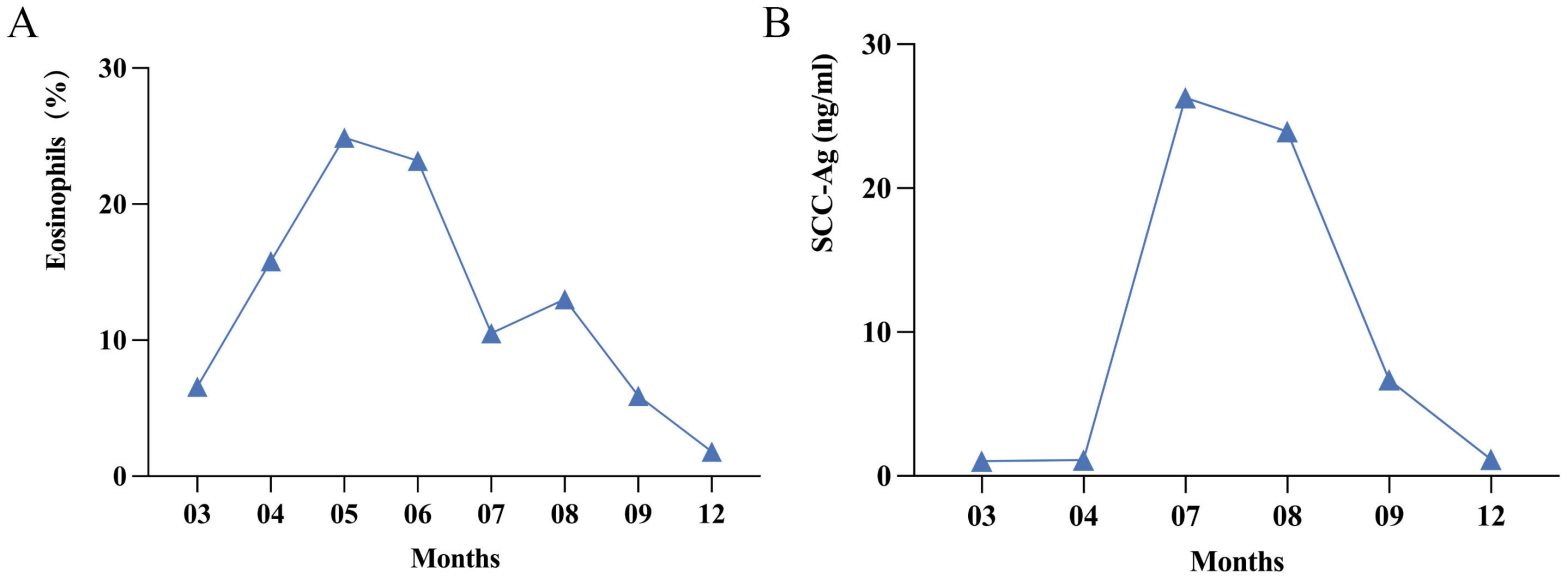
Changes in blood eosinophils and SCC-Ag levels. **(A)** Peripheral blood eosinophil percentage. **(B)** Serum SCC-Ag levels. SCC-Ag, squamous cell carcinoma antigen.

In June 2025, the patient initiated treatment with paclitaxel (100 mg, IV, on Days 1 and 8) in conjunction with cisplatin (25 mg, IV, on Days 1-3) and tislelizumab (200 mg, IV, on Day 8) on a 3-week cycle (q3w). During treatment, the patient developed generalized erythema with scaling, which was considered an immune-related cutaneous adverse event (**Figure 1B**); therefore, immunotherapy was discontinued. Starting from the third cycle, the patient received paclitaxel (150 mg, IV, on Day 1) plus cisplatin (25 mg, IV, on Days 1-3) every 3 weeks (q3w). A CT scan following the third cycle revealed a reduction in metastatic lymph node lesions in the left cervical and mediastinal regions compared with baseline (**Figure 2C**). However, his clinical condition deteriorated over the subsequent months; the treatment course was complicated by *Staphylococcus aureus* bacteremia and multiple episodes of SARS-CoV-2 infection, and the disease continued to progress. Interestingly, during treatment, the patient’s peripheral blood eosinophil count and squamous cell carcinoma antigen (SCC-Ag) levels showed an overall downward trend (**Figure 4**). In December 2025, he was readmitted because of sudden clinical deterioration. He initially presented with left-sided limb weakness and persistent vomiting, followed by disorientation, incoherent speech, and impaired attention. Repeat CT revealed a brainstem metastatic lesion (**Figure 2D**). Due to extensive metastases, deteriorating clinical status, and the absence of viable treatment alternatives, the patient was moved to palliative care and died within a few weeks.

## Discussion

This study reports a rare clinical case of an elderly male who presented with painless cervical lymphadenopathy accompanied by generalized pruritus and persistent peripheral blood eosinophilia. Despite a comprehensive evaluation, no primary site was identified, and the final pathological diagnosis was SMARCB1-deficient undifferentiated carcinoma. The patient experienced rapid disease progression shortly after receiving platinum-based chemotherapy combined with a PD-1 inhibitor and ultimately died from brain metastasis. This case integrates several uncommon and clinically challenging features, providing a unique perspective for exploring the biological behavior, diagnostic pitfalls, and therapeutic dilemmas associated with this highly aggressive malignancy.

CUP consists of a diverse array of metastatic cancers where the basic origin remains undetermined despite thorough diagnostic evaluation, accounting for approximately 2-5% of all cancers. In fact, even after positron emission tomography-computed tomography (PET-CT) or autopsy, the primary tumor cannot be determined in 20-50% of cases (14). Several potential mechanisms have been proposed. For example, the primary lesion may be too small to be detected with currently available diagnostic technologies (15). In addition, early dissemination may occur before the primary tumor becomes clinically detectable; the primary lesion may then be eliminated through immune-mediated clearance, whereas metastatic foci continue clonal growth (16). Overall, CUP is linked to an unfavorable prognosis, with a median OS of 7–11 months, a 1-year survival rate of <25%, and a 5-year survival rate of <10% (17). For unfavorable-risk CUP, both the National Comprehensive Cancer Network (NCCN) and the European Society for Medical Oncology (ESMO) guidelines recommend empiric chemotherapy based on platinum agents, taxanes, or gemcitabine, and encourage enrollment in clinical trials whenever feasible (18). Briasoulis et al. (19) reported an overall response rate of 38.7% with carboplatin plus paclitaxel, with a median OS of 13 months. A meta-analysis by Lee et al. (20) showed a median OS of 9 months in patients with CUP and suggested that platinum- or taxane-based regimens tended to be associated with improved survival compared with other chemotherapy approaches.

The treatment course in this case highlights the current therapeutic dilemma in managing SMARCB1-deficient CUP. The patient initially received an empiric CUP regimen (paclitaxel plus cisplatin) combined with a PD-1 inhibitor (tislelizumab), but the response was poor, with rapid disease progression, which may reflect multiple resistance mechanisms. First, SMARCB1 deficiency itself may confer broad drug resistance. Widespread epigenetic dysregulation can enhance cellular plasticity and contribute to aberrant DNA repair pathways and evasion of apoptosis (21). Studies have shown that inactivation of SWI/SNF chromatin-remodeling complexes is associated with altered sensitivity to certain chemotherapeutic agents, such as topoisomerase inhibitors, and may also influence the efficacy of immunotherapy by reshaping the tumor immune microenvironment (22, 23). In the present case, disease progression could not be controlled by chemotherapy alone after discontinuation of immunotherapy, underscoring the urgent need to develop targeted therapeutic strategies. Enhancer of zeste homolog 2 (EZH2) inhibitors have demonstrated preclinical activity in SMARCB1-deficient models, including malignant rhabdoid tumors, by inhibiting the histone methyltransferase EZH2 to counteract the reduced expression of tumor-suppressor genes and the upregulation of oncogenic pathways resulting from loss of SWI/SNF complex function (24). Tazemetostat, an EZH2 inhibitor, has received approval from the U.S. Food and Drug Administration for the treatment of SMARCB1-deficient epithelioid sarcoma. In addition, tazemetostat is under investigation in registered clinical investigations for tumors such as locally advanced SMARCB1-deficient sinonasal carcinoma (11, 25). However, this treatment was not administered during the patient’s clinical course. Several factors influenced this decision. First, tazemetostat was not readily available for this indication at our institution, and the patient did not have access to an appropriate clinical trial. Second, the disease progressed rapidly and was complicated by several serious adverse clinical events, resulting in a marked deterioration in the patient’s general condition. Consequently, the therapeutic window for initiating an investigational targeted therapy was very limited, and the patient was ultimately transitioned to palliative care. HH2853 is a dual EZH1/2 inhibitor, and the ongoing HH2853-G101 study is evaluating its activity in SMARCB1-deficient epithelioid sarcoma (26). In addition, immune checkpoint blockade has attracted particular interest. Ongoing or early-phase studies have evaluated dual immune checkpoint blockade with nivolumab plus ipilimumab (NCT04416568), combined inhibition of T-cell immunoreceptor with immunoglobulin and ITIM domains (TIGIT) and programmed death-ligand 1 (PD-L1) with tiragolumab plus atezolizumab (NCT05286801), and the combination of tazemetostat with nivolumab and ipilimumab (NCT05407441). Other investigational strategies aim to exploit vulnerabilities in cell-cycle regulation and DNA-damage-response pathways resulting from SMARCB1 loss, including Aurora A kinase inhibition and emerging synthetic-lethal approaches targeting DNA-repair pathways in selected SMARCB1-deficient tumor subtypes (27, 28). However, these strategies remain investigational, and the available evidence is derived primarily from early-phase clinical studies involving small sample sizes and biologically heterogeneous patient populations. Further validation in prospective, biomarker-driven clinical trials is therefore required. This case further suggests that for such highly aggressive malignancies, even when initial presentation is limited to regional lymph node metastasis, clinicians should remain vigilant for the risk of early central nervous system (CNS) dissemination. The fatal brainstem metastasis that developed in the late disease course highlights the potential need to consider CNS surveillance or prophylactic strategies early during treatment.

Another prominent feature of this case was marked and persistent peripheral blood eosinophilia accompanied by generalized erythematous rash-like lesions and pruritus. The precise mechanisms underlying TABE remain incompletely understood but may be related to cytokines secreted by tumor cells, such as interleukin (IL)-3, IL-5, and granulocyte-macrophage colony-stimulating factor (GM-CSF), which can specifically promote eosinophil proliferation, activation, and chemotaxis (29). Although TABE is overall uncommon in solid tumors, its presence is often associated with tumor burden, aggressiveness, and poor prognosis (30). In this case, eosinophilia occurred concomitantly with the cutaneous manifestations, and during the initial course of systemic therapy, the eosinophil count showed a declining trend in parallel with decreases in tumor markers such as SCC-Ag. This synchronous pattern suggests that the eosinophilia represented a tumor-driven paraneoplastic syndrome rather than an independent eosinophilic disorder or an allergic reaction. Activated eosinophils may influence the tumor microenvironment directly or indirectly by releasing cytotoxic granule proteins and pro-inflammatory mediators. On the one hand, this may exert antitumor immune effects; on the other hand, it may be more likely to promote tumor growth, invasion, and metastasis by inducing tissue injury, fibrosis, and angiogenesis (31). However, caution is warranted because the subsequent immune-related cutaneous adverse event may have been confounded with the pre-existing paraneoplastic skin manifestations, thereby increasing the complexity of clinical management.

This case has several limitations. First, as this is a single case report, it remains unclear whether the observed findings such as the lack of response to immunotherapy are generalizable. Second, next-generation sequencing (NGS) and more sensitive whole-body imaging modalities, such as PET-CT, might have potential value in identifying occult lesions and could reveal additional actionable genomic alterations, thereby providing a rationale for targeted therapy. In addition, due to practical constraints, we were unable to assess eosinophil chemotactic cytokines/mediators in the tumor tissue to directly substantiate a causal relationship with TABE. Finally, although immunohistochemistry demonstrated apparent complete loss of nuclear SMARCB1 expression in the tumor cells, the available section lacked evaluable non-neoplastic elements that could serve as internal positive controls, and no additional tissue was available for repeat staining. Therefore, this finding should be interpreted with appropriate caution.

## Conclusions

In summary, we report an exceedingly unusual and challenging case of SMARCB1-deficient undifferentiated carcinoma presenting as CUP predominantly with cervical lymph node metastases and marked TABE. Despite platinum-based chemotherapy combined with a PD-1 inhibitor, the disease progressed rapidly and resulted in fatal CNS metastasis, suggesting an aggressive biological behavior and limited benefit from current empiric treatment approaches. Future prospective studies should leverage its epigenetic vulnerabilities to develop more effective therapeutic strategies, while enhanced CNS surveillance and optimized supportive care may help improve clinical outcomes.

## Data Availability

The datasets used and/or analyzed during the current study are available from the corresponding author on reasonable request. Requests to access these datasets should be directed to jiangfenfen06@163.com.
